# Mechanical hysteroscopic tissue removal or hysteroscopic morcellator: understanding the past to predict the future. A narrative review

**DOI:** 10.52054/FVVO.13.3.026

**Published:** 2021-09-24

**Authors:** M Franchini, O Ceci, P Casadio, J Carugno, G Giarrè, G Gubbini, U Catena, M Chiara de Angelis, A Di Spiezio Sardo

**Affiliations:** Demetra IVF Center-Villa Cherubini, Florence, Italy; Second Unit of Obstetrics and Gynecology, Interdisciplinary Department of Medicine, University of Bari “Aldo Moro”, Bari, Italy; Division of Gynaecology and Human Reproduction Physiopathology Unit, DIMEC, IRCCS Azienda Ospedaliero-Universitaria di Bologna, Italy; Obstetrics, Gynecology and Reproductive Sciences Department. Minimally Invasive Gynecology Division. University of Miami. Miami, FL USA; Private practice at Palagi, Azienda Sanitaria di Firenze, Florence, Italy; “Madre Fortunata Toniolo” Hospital, Bologna, Italy; Division of Gynecological Oncology, Department for the Protection of Women’s and Children’s Health and Public Health, Fondazione Policlinico Universitario A. Gemelli IRCCS L.go A. Gemelli, Rome, Italy; Department of Public Health, University of Naples, Italy; Department of Public Health, University of Naples, Italy

**Keywords:** Hysteroscopic tissue removal system, morcellator, hysteroscopy

## Abstract

**Background:**

In recent years, the available evidence revealed that mechanical hysteroscopic tissue removal (mHTR) systems represent a safe and effective alternative to conventional operative resectoscopic hysteroscopy to treat a diverse spectrum of intrauterine pathology including endometrial polyps, uterine myomas, removal of placental remnants and to perform targeted endometrial biopsy under direct visualisation. This innovative technology simultaneously cuts and removes the tissue, allowing one to perform the procedure in a safer, faster and more effective way compared to conventional resectoscopic surgery.

**Objective:**

To review currently available scientific evidence concerning the use of mechanical hysteroscopic morcellators and highlight relevant aspects of the technology.

**Material and Methods:**

A narrative review was conducted analysing the available literature regarding hysteroscopic tissue removal systems.

**Main outcome measures:**

Characteristics of available mHTR systems, procedures they are used for, their performance including safety aspects and their comparison.

**Results:**

A total of 7 hysteroscopic morcellators were identified. The diameter of the external sheet ranged from 5.25 to 9.0 mm, optics ranged from 0.8 to 6.3 mm with 0o angle. The cutter device diameter ranged from 2.9 to 4.5 mm most of them with rotation and reciprocation.

**Conclusions:**

We conclude that the adoption of mHTR has shown to reduce operating time, simultaneously cutting and suctioning tissue fragments avoiding the need for multiple removal and reinsertions of the device into the uterine cavity as well as reducing the volume of distension media required to complete the procedure compared to using the hysteroscopic resectoscope.

## Introduction

Endoscopic gynaecologic technology has substantially evolved over the past two decades and numerous innovations have resulted in an increasing number of tools available for the diagnosis and treatment of uterine abnormalities.

Mechanical hysteroscopic morcellator (mHM) systems are an innovative minimally invasive surgical technology available to treat a large number of intrauterine pathologies both in the operative room as well as in the office setting (Vitale et al., 2020). Since April 2014, mHMs have been commonly known as mechanical hysteroscopic tissue removal (mHTR) systems to avoid potential terminology misunderstanding with laparoscopic power morcellator after the US Food and Drug Administration (FDA) issued a black box warning for laparoscopic electromechanical morcellators. Whilst the FDA conclude that there is no risk with the use of hysteroscopic morcellators, this is still being discussed and evaluated (Salazar and Isaacson, 2018; US Food and Drug Administration, 2014). We will present a narrative review demonstrating the evolution of mHTR systems for the management of intrauterine pathology.

## Methods

A literature search of relevant papers was conducted using the electronic bibliographic databases PubMed, Scopus, Embase, Science Direct, and the Cochrane Library. The strategies for electronic search were the following combined search: Hysteroscopic morcellator OR Hysteroscopic tissue retrieval system OR Hysteroscopic tissue removal system.

Titles and/or abstracts of studies retrieved using the electronic search strategy, and those from additional sources were screened independently by two review authors (MF, JC) to identify studies that potentially meet the inclusion criteria outlined above. The full text of these potentially eligible studies was retrieved and independently assessed for eligibility by other two review team members (ADS, MDA). Any disagreement over the eligibility of a study was resolved through discussion with a third collaborator (OC).

A manual search of the reference list of included studies was also performed in order to avoid missing relevant data. We searched for published (full-text studies and meeting abstracts) and unpublished studies (i.e., for which a registered protocol was available) from the aforementioned electronic databases. The results were compared and any disagreement was resolved by consensus.

## HISTORY

The term ‘morcellation’ was coined from the French word “morcellement” – to subdivide – “to fragment tissue in order to allow its extraction from a cavity”. Originally tissue morcellators were used in laparoscopic surgery for the removal of intra-abdominal organs (Nezhat et al., 1994). It is interesting to describe the work of an eminent French surgeon Dr. Jules-Émile Péan, who was the first to use the expression in medicine “Du morcellement appliqué à l’ablation des tumeurs” Leçons extraites du tome VII de Clinique Chirurgicale, Paris 1887. And even specifically aimed at the uterus: “Du morcellement appliqué à l’ablation totale de l’utérus dans certains cas de tumeurs fibreuses et cancereuses”.

The first manual laparoscopic morcellator was created by Kurt Semm in 1977 which featured a hand activated cutting device (similar to the current hysteroscopic morcellators, except for the lack of rotational movement). In 1988 the Serrated Edge Macro Morcellator “Moto-Drive” (Serrated Edge Macro Morcellator -SEMM-; WISAP Medical Technology Gmbh, Germany) was introduced into clinical practice with a diameter of 15 mm and was used to extract tissue from the abdominal cavity during laparoscopic surgery (Semm, 1976).

Further innovations were made leading to the creation of the Power Drive Macro (WISAP Medical Gumby Technology Gmbh, Germany) which introduced a tissue protection system to minimise accidental injury, which led to the first electromechanical morcellator created by Steiner in 1993. The electromechanical morcellation was faster and had a stronger rotational force compared to the existing manual activated device and quickly replaced it in clinical practice (Steiner et al., 1993).

In 1977, Dr. Lanny Johnson, an orthopaedic surgeon from East Lansing, Michigan, USA, together with Dyonics Corporation described the first morcellator that was used for intra-articular knee surgery (Macmull and Gupte, 2015). Over the years, arthroscopic shavers have been used in many arthroscopic joint procedures. These power-driven devices are designed to mechanically remove soft tissue such as synovium, fat pad, plicas, and ligament remnants during arthroscopic procedures. There are shavers to trim denser soft tissues such as meniscus, articular cartilage or glenoid labrum and finally shavers for removing bone. Currently, shavers are an imperative equipment in routine arthroscopic work (Chen et al., 2017).

In 1999, Dr Mark Hans Emanuel, a gynaecologist from Utrecht, Netherlands, who at the time was working in Haarlem, Netherlands, created with the support of Smith and Nephew Company, (Andover, MA, USA) the first generation of hysteroscopic shavers with dedicated mechanical blades. Since then, several mHTR systems have become available: TruClear ® (Medtronic, Dublin, Ireland), MyoSure ® (Hologic, Marlborough, MA, USA) and the Integrated Bigatti Shaver ® (Karl Storz, Tüttlingen, Germany). All mHTRs use mechanical energy to simultaneously cut and aspirate tissue. Recently, an innovative hybrid system the Symphion™ (Minerva Surgical Inc, Santa Clara, CA, USA) which offers automatic aspiration of tissue fragments resected with bipolar radiofrequency through a self-contained, recirculating fluid management system has also become available (Table I).

**Table I:** Characteristics of the devices currently available on the market.

	Truclear ^TM^ 8.0 System FDA 2005	Integrated Bigatti Shaver (IBS) FDA 2012 2018	Myosure System FDA 2009	Truclear 5C System FDA 2012	Synphion System FDA 2014	TruClear Elite FDA 2008	Omni Hysteroscope FDA 2018
Manufacturer	Smith & Nephew (Medtronic)	Storz	Hologic	Medtronic	Boston Scientific Minerva Surgical	Medtronic	Hologic
Hysteroscope							
Diameter (mm)	9.0	6.3	7.25 6.25	5.25	6.3	7.25 6.0	6.0 5.5
w/o outflow sheet (mm)	8.0	---	7.25 6.25	5.6	---	7.25 6.0	6.0 5.5
Optic Size (mm)	3.5	6.3	2.0	0.8	6.3	1.9	2.0
Optic System	ROD Lens	ROD Lens	ROD Lens	Fiberoptic	ROD Lens	ROD Lens	ROD Lens
Optic Device	0°	6°	0°	0°	0°	0°	0°
Cutting Device							
Outer Diameter (mm)	4.0 Disposable	4.5 Reusable	3.0 4.0 Disposable	2.9 Disposable	3.6 Disposable	4.0 2.9 Disposable	3.0 4.0 Disposable
Action	Rot/Recip	Rotation	Simoultaneous Rot/Recip	Rot/Recip	RF bipolar plasma resection	Rot/Recip	Simoultaneous Rot/Recip
Window closure	Operator to set	Automatic	Automatic	Operator to set	Automatic	Operator to set	Automatic

Mm: Millimeters; w/o: without; Rot: Rotation; Recip: Reciprocation; RF: radiofrequency

Lastly, a disposable system Aveta ® (Meditrina, Inc., San José, CA, USA) with high-speed mechanical oscillation mechanism, received 510(k) premarket notification in May 2020.

### Structural design

mHTR systems were developed in an attempt to overcome the limits of traditional hysteroscopic resectoscopic procedures such as the risks of complications arising from the use of electrosurgical energy and thermal injury based on a non-cautery- dependent mechanical action. Furthermore, the ability to simultaneously cut and aspirate tissue optimises specimen removal and more importantly avoids the need to use additional instruments or to dredge the uterine cavity (Noventa et al., 2015; Hamidouche et al., 2015; Vitale et al., 2017).

Most of mHTR systems have a similar structural design consisting of a power control unit with dedicated software, footswitch, hand piece, hysteroscope and cutting blades (Singh et al., 2009).

#### Power Control Unit

An electric motor is located inside the Power Control Unit (PCU). A foot pedal activates the motor that drives the blade inside the hysteroscope. The PCU, is connected to the blade via a flexible drive cable, having the capacity to rotate and/or reciprocate the blade at an adjustable speed that is measured in revolution per minute (rpm). A digital display of the Control Unit shows the function mode (rotation and/or reciprocation), speed (rpm) and surgical time corresponding to the blade working time. Some mHTR systems sense the type of blade installed in the connected hand piece and automatically set the appropriate speed of the cutting blade (based on a pre-set program).

#### Footswitch

The foot pedal controls activation and deactivation of the motor which powers the morcellator in the blade. Depending on the different model, the footswitch allows pre-setting the mode of function of the blade (rotation, or rotation with reciprocation) with a dedicated button. Another button on the foot pedal helps to set, before starting the procedure, the window of the blade in the closed position (window lock). Pressing the button, the inner blade slowly turns until the window is closed.

#### Hand-piece

The hand-piece drives the surgical blades and provides manual control of the suction flow. Since the blade is placed in the operative channel of the dedicated hysteroscope, it is recommended that the handpiece should be held with the dominant hand (“pistol grip”). The connecting suction device is used to simultaneously retrieve the chips out of uterine cavity during the procedure.

#### Hysteroscope

A wide range size of rigid hysteroscopes with an offset proximal eyepiece are designed to accommodate the blades within a working channel. Dedicated continuous flow hysteroscopes utilise fibers or rod lenses for visualisation and are compatible with a custom-designed or generic fluid management system (Table I).

#### Shaver blades

Different diameter motorised blades with wide range of window sizes have been developed by the different companies. All blades access the uterine cavity through a straight working channel of the dedicated hysteroscope. The blade consists of an outer hollow sheath and an inner hollow rotating/cannula with corresponding windows for simultaneous suction and cutting. The inner tubes create negative pressure and absorb the tissue in proximity to the windows. The cutting blade can easily penetrate into the tissue and prevent ejection of tissue from the cutting window during closure. Since the tissue needs to be introduced into the opening, the speed of the cutting blade needs to leave enough time for tissue fragments to enter. The blade is connected to the hand-piece and also to a vacuum source which aspirates resected tissue through a side-facing cutting window in the device’s outer tube. Distension fluid and resected tissue are transported from the blade window to a tissue trap and vacuum canister via a tube protruding from the proximal end of hand piece. Recently, two manual HTR devices MyoSure Manual ® (Hologic, Marlborough, MA, USA) and ResectrTM Tissue Resection (Boston Scientific, Marlborough, MA, USA) have become available. Manual control enables physicians to perform tissue resection by squeezing and releasing the handle with their finger.

#### Window lock

A window lock stops the blade window in the closed position before and following its activation. This is a very useful function to keep the uterine cavity distended. Most mHTR systems have a pre- set window lock. When the window is not pre-set or previously locked, before beginning the procedure, the window can be closed, pressing the button of the footswitch, either before or after the insertion of the device in the uterine cavity. When a blade is changed, this needs to be verified or reset.

#### Irrigation and suction system

Normal saline solution is the most commonly used distension media with mHTR systems. Since an accurate control of intracavitary pressure and fluid balance is crucial to minimise the risk of fluid intravasation syndrome the saline solution must be delivered using an electronically controlled irrigation pump and suction device system.

Therefore, common to every mHTR systems is the use of automated fluid management systems that continuously measure the distending media input and output, the intrauterine pressure, and the fluid deficit volume. The fluid management systems allow an adequate visualisation of the intrauterine cavity during the procedure. An integrated vacuum suction provides a negative pressure through the central cylinder of the blade and brings the tissue fragments into the cutting window. As the blade rotates, the tissue is cut, and is instantly aspirated through the central tube and is collected in a suction trap.

A reliable irrigation system is crucial to maintain a clear view into the uterine cavity during the procedure (Vitale et al., 2020).

### Clinical practice

In recent years, the available evidence demonstrated that mHTR systems are a safe and effective minimally invasive alternative to conventional operative hysteroscopy in the treatment of intrauterine pathologies such as endometrial polyps, uterine myomas and removal of placental remnants among other gynaecological conditions. The introduction of miniaturised scopes such as the TruClear5C hysteroscope of 5.25 mm, the TruClearTM Elite Mini hysteroscope of 6.15 mm, the Omni hysteroscope of 5.5 mm and the mini Bigatti shaver (IBS) of 6.3 mm, has allowed the use these new smaller devices in an office setting. A randomised controlled trial conducted by Smith et al. (2014), included 121 women undergoing hysteroscopic polypectomy and demonstrated that intrauterine morcellation was less painful and more acceptable to women when compared with traditional bipolar resectoscopy for the removal of endometrial lesions in an office setting. These data favour the use of these new devices in an office setting.

The working mechanism of the mHTR systems is very simple. Once the cutting blade window is placed in close contact with the lesions to be removed, this new technology simultaneously cuts and aspirates the tissue improving visibility and reducing the need for multiple removal and insertions of the device from the uterine cavity (Noventa et al., 2015). The speed of tissue removal depends on the contact time of the cutting window with the pathology, the consistency of the tissue and the speed with which the blade cuts and aspirates the tissue. The advances of surgical technology and the desire of always creating better and safer technology that drives close collaboration between clinicians and the industry has allowed an improvement in the quality of the mHTRs resulting in the persistent creation of newer and safer versions of these devices.

#### Polypectomy

**Figure 1:**
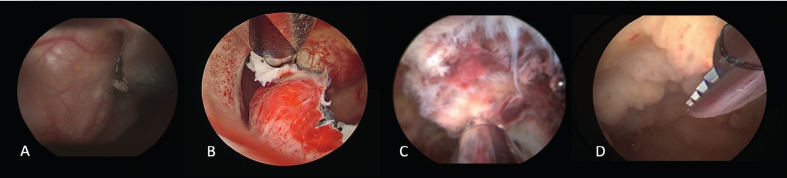
— Different uses of mechanical hysteroscopic tissue removal systems: A. endometrial polyp removal, B. myomectomy, C. removal of placental remnants, D. targeted endometrial biopsy.

Multiple trials have shown the suitability of mHTR systems for endometrial polyp removal. (Fig. 1A) Hamerlynck et al. (2011) have reported successful hysteroscopic removal of 278 polyps without complications in a retrospective descriptive study. Studies by Emanuel and Wamsteker (2005) and van Dongen et al. (2008) have shown that mHTR is significantly faster for the removal of polyps compared with conventional loop resectoscopy, with equivalent removal of pathology when performed in the operating room. Smith et al. (2014) and Pampalona et al. (2015) highlighted that in an office setting mHTR polypectomy had a shorter procedure time and higher complete removal of the pathology compared with bipolar electrode. Since patient acceptability and procedural pain were linked to the duration of hysteroscopic surgery, the reduction in the total operative time is of high clinical significance for patient acceptability in an office setting (Litta et al., 2008; Bettocchi et al., 2004). Ceci et al. (2019) demonstrated the feasibility and effectiveness of mHTR for the removal of large sized endometrial polyps in an office setting. AlHilli et al. (2021) highlighted that the cumulative incidence of polyp recurrence after 2 years was 4.5% with resectoscopy and 0.8% with mHTR. When polypectomy was performed in an office setting with a small sized mHTR, Ceci et al. (2020) found after one-year, that the recurrence rate of the polyp was higher at 10.4% with bipolar electrode compared to 7.1% with mHTRs, but the difference was not statistically significant (p= .99).

Removal of endometrial polyps using mHTR provides adequate tissue for histological diagnosis despite the effects of tissue fragmentation. The visibility of the epithelial component of the specimen remained unchanged, thus allowing a precise complete pathologic diagnostic assessment on all fragments of cytologic features, and of the stromal vascular component, that was never compromised by morcellation. As such, tissue integrity is a constant feature with all mHTR systems (Franchini et al., 2015).

#### Myomectomy

The rate of complete removal of myoma with the mHTR systems was found to vary considerably depending on the size and type of myoma. Studies have tried to evaluate the cut off values for volume and diameter of myoma, in order to obtain the best surgical outcome with the lowest possible risk using the mHTR (Friedman et al., 2018).

Arnold et al. (2016) demonstrated that the removal of the entire pathology ranges from 90% for myomas smaller than 2 cm to 48% for myomas larger than 4 cm. Hamidouche et al. (2015) found no significant difference in the rates of complete removal of the pathology in a single procedure between the mHTR (64%) and the bipolar resectoscope (69%) for types 0, 1 or 2 submucous myomas.

In a recent published meta-analysis, no statistically significant difference in length of surgery was observed in case of submucous leiomyomas when compared to traditionally resectoscopy (Vitale et al., 2017).

Bigatti et al. (2014) described advantages of the shaver technique, allowing treatment to occur in a single step procedure, 93.5% of myomas less than 3 cm when type 0 or 1 and 62.5% of type 2 myomas. In 2017, Liang et al. demonstrated in an observational study the feasibility of mHTR for the removal of type 2 myomas combining mHTR morcellation with cold scissors and grasping forceps in an operating room (Liang et al., 2017). Munro (2016) reported a new technique designed to expand the capacity of mHTR systems to completely remove deep type 1 and 2 myomas. Using a bipolar radiofrequency needle to allow the “release” of the lesions, they improved access of the intrauterine morcellator to myomas that previously were inaccessible. However, using this innovative technique, there was no reduction in surgical time noted, but the use of mHTR did minimise myometrial trauma preserving the pseudocapsule (Fig.1 B)

In 2017, Vitale et al demonstrated in a systematic review that mHTR systems are very effective for the excision of submucous type 0-1 myomas, but less efficient for type 2 submucous fibroids and also showed similar resection rates between the two, at the time available, FDA-approved hysteroscopic morcellators (TruclearTM and Myosure ® ) (Vitale et al., 2017).

A meta-analysis of two RCTs showed significantly less fluid deficit among women who underwent myomectomy treated with morcellation (With morcellation device=36.16 mL; 95% CI, -60.66 to -11.67) without heterogeneity (I2=0%) comparing to resectoscopy (Shazly et al., 2016). This reduced risk of intravasation could be due to the reduced time of the procedure that it is usually associated with the use of mHTR systems. However, the 2 RCTs included in the meta-analysis were both based on treating polyps, not fibroids. There was also a significant difference in procedure time between hysteroscopic morcellation and resection favouring the former. For the subgroup of fibroids, the difference was non-significant, but there seems to be a trend towards morcellation. However, on scrutinising the two studies focusing on the treatment of fibroids (Hamidouche et al., 2015; Emanuel and Wamsteker, 2005), there is an underrepresentation of type 2 fibroids: in the Hamidouche study only 29% in the morcellation group versus 41% in the resection group. Moreover, in the Emanuel study type 2 fibroids were excluded from the analysis.

#### Retained products of conception (RPOC)

The frequency of patients with retained products of conception (RPOC) after any form of pregnancy termination is increasing (Van den Bosch et al., 2008). Traditionally, the surgical approach for the removal of RPOC was blind dilation and curettage (D&C), but it is well known that this procedure is associated with higher risk of complications such as infections, intrauterine adhesion formation and uterine perforation (Pacheco et al., 2019).

mHTR systems represent an effective alternative to blind sharp or suction curettage for the management of RPOC (Fig.1 C) (Ikhena et al., 2016; Rein et al., 2011). When using mHTR, the operator is able to selectively remove products of conception under direct visualisation, causing minimal damage to the endometrium and reducing the risk of post-operative adhesion formation (Ansari et al., 2018).

Since Hamerlynck et al. (2013) first described the use of mHTR for the management of RPOC, the advantages of mHTR with complete removal of RPOC has been confirmed by several case series (Mallick and Middleton, 2017; Sutherland and Rajesh, 2018; Capote et al., 2018).

In 2016, Hamerlynck et al. confirmed in a randomised controlled trial that the mHTR is a faster alternative to loop resection. Both techniques are safe and show high rates of complete removal and tissue availability with 3% of de novo intrauterine adhesion formation (Hamerlynck et al., 2016).

Data on reproductive and obstetric outcomes after HTR and resection have been recently published by van Wessel et al. (2020). The mean time from procedure to conception after removal of RPOC was similar at 14 weeks with HTR and 15 weeks with loop resection. The live birth rate was higher in HTR (88.9%) compared to resection (68.2%), although the difference was not statistically significant.

### Targeted endometrial sampling

The use of mHTR systems has been proposed to mechanically remove targeted endometrial tissue without damaging the surrounding healthy endometrium. The idea is to perform with mHTR a “curettage” under direct visualisation of the uterine cavity (“visual D&C”) replacing the classic blind D&C (Fig.1D).

Rosenblatt et al. (2017) highlighted the ability of mHTR to collect a targeted large quantity of tissue for accurate and detailed histological evaluation, compared to the samples obtained with D&C in women with postmenopausal bleeding.

Franchini et al. (2013) demonstrated that the removal of endometrial polyps using mHTR provides adequate tissue for histological diagnosis despite the effects of tissue fragmentation but without any thermal artifact in tissue samples submitted for histological evaluation.

In contrast, Lindheim et al. (2019) questioned the ability to accurately perform histological reads of specimens collected using mHTRs. In a small study they found that mHTRs may affect the pathologist’s ability to diagnose the specimen. In their study, up to 30% of malignant specimens were overdiagnosed and up to 20% underdiagnosed.

Since specimens obtained with mHTRs are immediately suctioned and collected in a trap, a target sampling could be obtained separately in a target trap improving the quality of the specimen for pathological analysis.

“Visual D&C” with target sampling could represent an alternative for young patients diagnosed with endometrial hyperplasia/cancer who want to preserve their fertility. However, further studies are needed to confirm the feasibility of this indication. It is important to highlight the diagnostic value of collecting the endometrial biopsy under direct visualisation, which allows targeted biopsy, avoiding, when possible, blind intrauterine procedures and minimising the risk of failing to diagnose focal endometrial pathology.

### Further indications

#### Removal of uterine septum

Simons et al. (2011) proposed that mHTR systems might be a safe and effective alternative for resectoscopy in removing avascular uterine septa and that it may cause fewer complications such as fluid overload or thermal injuries. In addition, it is associated with reduced operating time, and removal instead of transection of the septum may lead to less intrauterine adhesion formation.

#### Overcoming severe cervical stenosis

Salari et al. (2016) in a video article demonstrated the use of mHTR systems to safely gain access into the intrauterine cavity in patients with severe cervical stenosis minimising the potential risk of uterine perforation and false tracks formation. At the present time, further research is needed to confirm this finding.

## Comparison

### In vivo

To the best of our knowledge, until now, no study has been performed comparing the tissue resection characteristics of the different commercially available mHTR systems. Nevertheless, Shazly et al. (2016) in a meta-analysis of four randomised clinical trials and three retrospective observational studies showed similar fragmentation rates between different mHTR systems and similar complete resection of the pathology rates with smaller fluid deficit compared to hysteroscopic resectoscopy and concluded that using any of the available mHTR systems produce similar clinical outcomes.

### In vitro

A randomised independent in vitro comparison of two FDA approved mHTR systems has been performed to assess the tissue resection characteristics of the different blades (TruClearTM INCISOR 2.9 (TI), TruClearTM Incisor Plus (TIP), or TruClearTM Ultra Plus (TUP) and MyoSure ® Lite (ML), MyoSure ® Classic (MC), or MyoSure ® XL (MXL)) (Meulenbroeks et al., 2017). Using a surrogate (umbilical cord) polyp, the study has shown that although the larger TIP, MC, and ML devices were significantly faster than the TI for removal of one polyp, only the TIP was consistently faster than the TI for consecutive removal of polyps. The performance of the ML decreased significantly during removal of three consecutive tissue samples, making it slower than the TIP with a similar window size in the third run. For removal of myoma tissue, the resection rate of the TUP was significantly higher than that of the MXL, and the resection rate of the MXL decreased with increasing myoma volume. On the contrary, another in vitro manufacturer sponsored study has reported that MXL is as 2.5 times faster than TUP for the excision of calcified fibroids.

### Cost evaluation

Traceable items are the major factor identified to monitor the cost of hysteroscopic procedures. Costs of mHTRs are offset by the cost of the single-use blade or by the cost represented by the damage caused to the reusable blades during its use (Franchini et al., 2018).

Since all available mHTR systems have shown both a similar tissue fragmentation and complete resection rate, the choice of one device over another does not yield a significant difference in clinical outcomes but it could incur in different cost to perform the procedure. An increased market competition should deliver what customers need at a more affordable price.

### Learning curve

Since the first comparative study on mHTR systems was published in 2005, the use of mHTR has been recognised as a safe and easy to learn alternative to hysteroscopic resection with procedures completed in almost one third of the usual time (Emanuel and Wamsteker ., 2005).

van Dongen et al. (2008) in a pilot randomised controlled trial conducted to evaluate the learning curve of physician residents in training using mHTR systems, demonstrated a very short learning curve. They have found that only 3% of the mHTR procedures could not be completed by the residents themselves, whereas 17% of resectoscopic procedures needed the intervention of senior surgeons to complete the procedure (P   0.001). Pampalona et al. (2015) have also showed similar data in an office setting. Residents required supervision by the senior surgeon in the use of mHTR only during the first procedures with inability to complete procedures in only 8% of cases. Furthermore, senior surgeons and residents experienced a comparable shortening of the procedure time independently from their skills in operative hysteroscopy.

Since the amount of tissue removed with a mHTR blade is a function of how much contact the cutting window maintains with the pathology and how quickly the blade can cut and aspirate the tissue, the potential benefits of mHTR seems to be the automatisation and the simplification of the surgical technique. In hysteroscopic surgery, the promise of simplifying complex manual control of resectoscopic tissue resection, which requires to move the loop back and forth squeezing and releasing the handle, allows for less reliance on the surgeon’s operative hysteroscopic skills.

### Limits and complications

As per the FDA’s MAUDE (manufacturers and users device experience) database, mHTR systems are associated with fewer life-threatening complications such as fluid overload, uterine perforation, and bleeding when compared to resectoscopy (Haber et al., 2015). The prevalence of complications is extremely low (0.02% in hospital; 1.6% office) and less frequent than the complication rate reported with the use of with traditional hysteroscopic resection (Noventa et al., 2015).

The limitations to the use of mHTR systems remain to be fully elucidated. The risk of cancer cell dissemination within the peritoneal cavity during hysteroscopic procedures in patients with endometrial cancer, is still under discussion (Chang et al., 2011). Nevertheless, FDA Safety Communication stated that mHTR systems, when used in accordance with their intended use, have no risk of spreading unrecognised malignant cells (US Food and Drug Administration, 2014).

## Conclusion

In the presence of intrauterine pathologies requiring surgical treatment, mHTR represents a great minimally invasive choice for the complete removal of intracavitary lesions with adequate tissue quality for histologic analysis and without damaging the surrounding healthy endometrium.

The adoption of mHTR has shown to reduce operating time, simultaneously cutting and suctioning tissue fragments avoiding the need for multiple removal and insertions of the device into the uterine cavity as well as reducing the volume of distension media required to complete the procedure compared to using the hysteroscopic resectoscope.

It is our opinion that although the mHTR are gaining an important role in clinical practice, the hysteroscopic resectoscope will continue to be a very important tool for the management of patients with intrauterine pathology.

Based on the current data, all commercially available mHTR systems show comparable properties and they offer a fast, precise, safe, and easy to learn alternative to conventional resectoscopic surgery (Shazly et al, 2016; Yin et al., 2018; Li et al., 2017). Therefore, gynaecologists should be encouraged to choose mHTR systems when available and to always avoid performing intrauterine blind procedures.
